# The weepy cry – short neural signal bursts in intraoperative neuromonitoring

**DOI:** 10.1007/s00423-024-03240-z

**Published:** 2024-03-22

**Authors:** Philipp C. B. Munk, Mick E. Merkelbach, Wolfram Lamadé

**Affiliations:** 1 Ruprecht-Karls-Universität Heidelberg, Heidelberg, Germany; 2 BG Klinikum Murnau, Murnau, Germany; 3 Helios Klinikum Pforzheim, Pforzheim, Germany

**Keywords:** Recurrent laryngeal nerve palsy, Intraoperative neuromonitoring, Nerve conduction studies, In-vitro

## Abstract

**Purpose:**

This study aimed to establish an in-vitro alternative to existing in-vivo systems to analyze nerve dysfunction using continuous neuromonitoring (C-IONM).

**Methods:**

Three hundred sixty-three recurrent laryngeal nerves (RLN) (N_(pigs)_ = 304, N_(cattle)_ = 59) from food industry cadavers were exposed by microsurgical dissection following euthanasia. After rinsing with Ringer's lactate, they were tempered at 22 °C. Signal evaluation using C-IONM was performed for 10 min at 2 min intervals, and traction forces of up to 2N were applied for a median time of 60 s. Based on their post-traumatic electrophysiological response, RLNs were classified into four groups: Group A: Amplitude ≥ 100%, Group B: loss of function (LOS) 0–25%, Group C: ≥ 25–50%, and Group D: > 50%.

**Results:**

A viable in-vitro neuromonitoring system was established. The median post-traumatic amplitudes were 112%, 88%, 59%, and 9% in groups A, B, C, and D, respectively. A time-dependent further dynamic LOS was observed during the 10 min after cessation of strain. Surprisingly, following initial post-traumatic hyperconductivity, complete LOS occurred in up to 20% of the nerves in group A. The critical threshold for triggering LOS was 2N in all four groups, resulting in immediate paralysis of up to 51.4% of the nerves studied.

**Conclusion:**

Consistent with in-vivo studies, RLN exhibit significant intrinsic electrophysiological variability in response to tensile forces. Moreover, nerve damage progresses even after the complete cessation of strain. Up to 20% of nerves with transiently increased post-traumatic amplitudes above 100% developed complete LOS, which we termed the "weepy cry." This time-delayed response must be considered during the interpretation of C-IONM signals.

## Introduction

Several studies identified surgical interventions such as thyroidectomy or similar procedures among the most common causes of RLN paralysis [1–3]. Different risk factors for an unfavorable surgical outcome have been identified. These include Graves' disease, revision surgeries, hematoma exploration, and malignancy. Likewise, surgical volume is an important aspect. Experienced surgeons generally achieve comparatively better surgical outcomes, particularly those with expertise in head and neck surgery [1, 4]. Thus, a minimum amount of thyroidectomies has been suggested to improve individual surgical training, thereby reducing RLN palsy rates [5]. However, other causes of vocal fold paresis unrelated to direct RLN injury, such as dislocation of the arytenoid cartilage after intubation, have also been described [6].

Localization of the RLN is regarded as the gold standard in thyroid surgery to avoid nerve lesions [4, 7, 8]. During neural mapping, intraoperative neuromonitoring (IONM) aids in the visual detection of nerves [8, 9]. Identification rates of up to 99.3% have been reported when supported by neuromonitoring, as opposed to 90% without it [10]. In addition, IONM facilitates the assessment of preoperative compared with postoperative nerve function. As such, it may guide decision-making in case of signal deterioration, thereby reducing transient nerve palsies [4, 8, 9]. The auditory and visual display of signal strength can alert surgeons to refrain from harmful procedures such as nerve stretching. Consequently, it can be argued that IONM is particularly useful in training surgeons to recognize risky behaviors [8, 9]. Thus, this technique has been shown to improve intraoperative and postoperative management of RLN lesions [9]

Intraoperative injury to the RLN alters the amplitude and latency of a neural signal, as measured by the IONM device. This enables the detection of minuscular changes that might otherwise be overlooked [9–11]. Amplitude reductions of more than 50%, particularly if combined with an additionally increased latency of more than 10%, indicate nerve damage. In combination, they have shown a sensitivity of 100% and a positive predictive value of 97.7% for the diagnosis of RLN injury [10, 12–14]. The decline in amplitude correlates with the number of axons lost, with a complete LOS representing total axonal injury and conduction block [15].

RLN injury is accompanied by a fall in amplitude due to axonal degeneration causing focal demyelination, a process defined as neurapraxia [10, 15, 16]. Usually, this leads to a gradual reduction in amplitude, which is reversible if detected early, provided that the anatomical continuity of the nerve is preserved [9, 10]. However, chronic or repeated injury (traction, compression, thermic), may eventually progress to complete LOS and more severe axonal injury. This phenomenon has been described as a double crush injury [16, 17]. Nerves are more likely to develop compressive neuropathy if they have previously sustained compressive lesions. The additive effect of multiple subthreshold injuries combines to form a suprathreshold lesion, disrupting axonal traffic [16]. Previous research investigated tensile injuries in an in-vivo environment [18]. Therefore, we aimed to develop an in-vitro alternative to this approach.

## Material and Methods

Three hundred sixty-three RLN were dissected from the pharynges of pigs (*Sus scrofa domesticus,* *N* = 170, 5 – 6 months old) and cattle (*Bos taurus,* *N* = 30, 20 – 40 months old). The animals were bred for the food industry, and the pharynges were harvested immediately after euthanasia.

### Sacrificing procedure

All animals were sacrificed at the abattoir in accordance with the German Animal Welfare Act [19]. Per the law, pigs received electric shocks of 297 V (2.0 A) at a frequency of 60 Hz for 4 s to the head and heart. This induced an epileptic seizure, stunning them. Subsequently, the aorta and heart were severed via a lateral neck incision, resulting in death by blood loss. In contrast, the cattle were not subjected to an electric shock, but a bolt shot into the animal's forehead. The pressure vibrations of the shot, combined with the penetration of the cerebral cortex, induced a stunning concussion. However, since the brainstem was unaffected, this had no lethal effect. An incision in the neck severed the aorta and heart, resulting in the animal's death. Afterward, the pharynx-lung-heart complex of both species was excised by the butchers. After being suspended for two to three minutes to drain the blood, it was examined by the veterinarian. Finally, lungs and hearts were removed, the pharynges separated by gender, and stored in a transport box along with gloves filled with warm water, keeping the overall temperature at approximately 20 °C.

### Timing of the trials

The procedure started with the euthanasia of the animal (T0) at the abattoir. The pharynges were then transported to the laboratory, and the RLN was dissected (T1). Next, the nerve was stimulated by IONM to determine if a positive signal response could be detected. If so, the nerves entered the trial phase (T2). After trial completion, or if a nerve did not produce a positive signal upon stimulation, they were discarded (T3).

### Nerve preparation

A sample quality score (SQS) was established for every side (left/right) of the pharynges. These ranged from one to six, with one being the best and six the worst possible grade. Grading criteria are included in Table 1.

**Table 1:** Grading criteria used to evaluate the used pharynges

Points	Condition
I	• The RLN is fully covered by its surrounding tissue
I	• No hematomas are visible
I	• No blisters caused by electricity can be observed
I	• The trachea remains intact
I	• The esophagus contains no incisions
I	• The surrounding neck muscles are present and unharmed

Grade 1: 6 Points, Grade 2: 5 Points, Grade 3: 4 Points, Grade 4: 3 Points, Grade 5: 2 Points, Grade 6: 0 – 1 Points

Initially, a pharynx was removed from the warmed storage box, and placed in the lateral position on the laboratory table, where its temperature was measured. Since the lamina superficialis of the cervical fascia, the *musculi platysma*, *sternohyoidei*, and *sternomastoidei* were already removed by the butchers; the dissection focused on the lateral side of the pharynx. Beginning in the now-connected *Trigonum caroticum*, the superficial connective tissue was removed, and the RLN was located in the groove between the trachea and esophagus. The nerve was then carefully separated from its surrounding tissue. Depending on the highly individual condition of the specimen, the nerve was exposed over approximately ten to thirty centimeters while remaining within the pharyngeal tissue complex. Finally, the nerve was irrigated with Ringer's lactate, and its length and diameter were measured.

## Conduction of the experiments

**Fig. 1:**
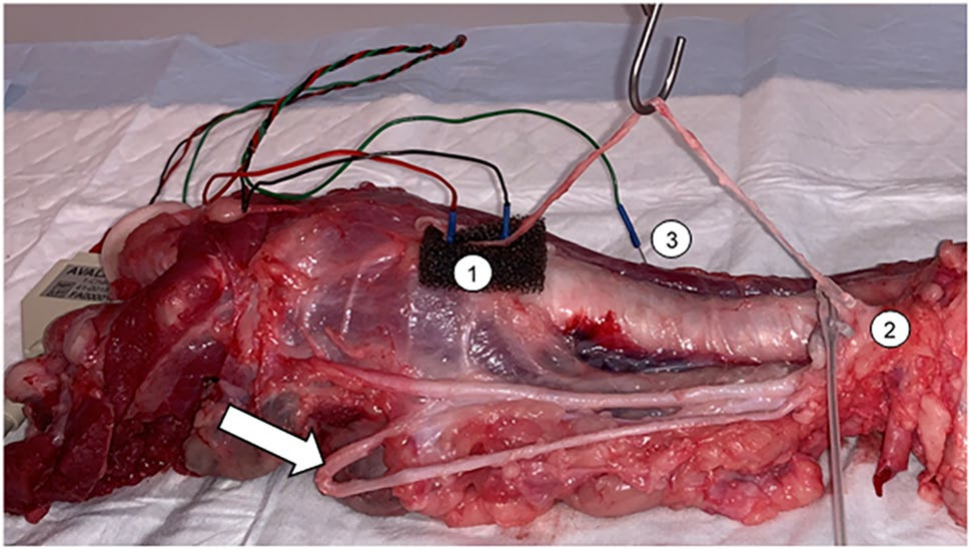
Experimental setup of a dissected RLN in a pigs’ pharynx **1:** Needle electrodes, **2:** Saxophone electrode, **3:** Grounding electrode, **Arrow**: Vagus nerve

First, the cranial part of the RLN was placed on a piece of foam to electrically insulate it from the surrounding tissue. Then, needle electrodes (Dr. Langer Medical GmbH) were inserted through the nerve into the foam, while the grounding electrode was introduced into a nearby muscle. A saxophone electrode (Dr. Langer Medical GmbH) was attached caudally to the needle electrodes. Next, the electrodes were connected to the neuromonitoring device (Avalanche^XT^ Neuromonitor © Dr. Langer Medical GmbH), and the saxophone electrode stimulated the nerves with 1 Hz pulses at 0.5—5 mA (Fig. 1). An initial baseline assessment of the electrophysiological properties of the nerve was performed. If the nerve displayed no response to electrical stimulation, it was discarded, and the contralateral side was dissected. However, if the nerve did respond to electrical stimulation, the baseline signal parameters of amplitude, latency, threshold current, and supramaximal voltage were determined. The latter two parameters were established by starting nerve stimulation at 0.1 mV and gradually increasing the stimulation in 0.1 mV increments. Subsequently, the nerve was positioned on a spring scale midway between the saxophone electrode and the needle electrodes, applying tensile forces between 0.5 N – 2 N for sixty seconds.

Immediately after strain exposure (Min 0) and for the following ten minutes (Min 2 – 10), the electrophysiological properties were recorded using C-IONM in two-minute intervals. If the nerve still exhibited a positive signal ten minutes later, it was subjected to another trial. In the case of LOS, the time difference (TD) between LOS and the application of tensile forces (Min 0) was documented. The nerve was then checked every five minutes to assess for spontaneous signal recovery. If it did recover, the signal parameters were documented at two-minute intervals for ten minutes, as well as the time difference between LOS and recovery.

### Data processing and statistical analysis

Data was recorded into an Excel spreadsheet and further processed using various algorithms written in Python version 3.8.10. The libraries Openpyxl version 3.0.7, Pandas version 1.3.5, Matplotlib version 3.5.1, SciPy version 1.7.3, scikit-learn version 1.3.2, statsmodels version 0.14 and NumPy version 1.21.5 were used for further analysis and graphics creation.

For a more accurate comparison of the measured parameters, the absolute values of the parameters were converted to their percentage changes. Here, the first measured value before the nerve was subjected to strain was used as the baseline value corresponding to 100%. The values of the following time points (Min 0 – Min 10) were calculated as the percentage deviation from it. Amplitude reductions are associated with the number of lost axons [15]. Thus, the nerves were divided into four categories according to their amplitude immediately after strain: Group A (signal above 100%), Group B (signal between 100 and 75%), Group C (signal between 75 and 50%), and Group D (signal below 50%).

Statistical analysis was conducted using Python, assuming a value of p < 0.05 as statistically significant. The data were tested for normal distribution using the Shapiro-Wilks test. An independent-student T-test, the Wilcoxon Signed-Rank test, the Mann–Whitney U test, or Spearman’s Rank-Order correlation were used where appropriate. Multivariate regression models were used to control for confounders. For the prediction of amplitude values, the covariates Sample state, Nerve length and diameter, time difference (T1 – T2), time in use (T2 – T3), and temperature change were inserted into a generalized linear model. The selection of covariates was theory-driven.

## Results

### Total dissected and participating nerves

Three-hundred sixty-three RLN were harvested from 200 pharynges, resulting in 304 (83.7% of the total dissected) pig nerves and 59 (16.3%) cattle nerves. An overall median SQS of four (range: 1 – 6), as well as a median time difference between animal death (T0) and nerve dissection (T1) of 3:44 h (range: 1:10 h – 9:10 h), was observed. Furthermore, a median time in trial use (T2 – T3) of 16 min (range: 0 min – 6:31 h) was measured, as was a median temperature change (T2 – T3) of 0.4 °C (range: 0.7 °C – 2.5 °C). Of all sampled nerves, 46% (*N* = 167) yielded a positive signal upon stimulation, representing the majority (81%, *N* = 47) of all cattle and 39% (*N* = 118) of all pig nerves.

Eighty-seven RLNs were used in 128 experimental trials in this proof-of-concept study. The remaining nerves were preserved for a follow-up study, based on the findings of this work. 66.7% (*N* = 58) of the used nerves were obtained from pigs and 33.3% (*N* = 29) from cattle. With 69% (*N* = 60), most used nerves originated from female animals, while 31% (*N* = 27) were male animals. 47.1% (*N* = 41) of all used nerves were left-sided, and 52.9% (*N* = 46) were right-sided. The used nerves exhibited a median SQS of three (range: 1 – 6) and a median time difference (T0 – T1) of 3:01 h (range: 1:10 h – 8:55 h). The median time in trial use (T2 – T3) was 70 min (range: 1 min – 6:31 h), with a median temperature change of 0.5 °C (range: -1.7 °C – 8 °C). Finally, a median diameter of 1.5 mm (range: 0.7 mm – 6 mm) and a median length of 17 cm (range: 8 cm – 81 cm) were measured (Table 2).

**Table 2:** Overview of the physical characteristics of all participating RLN

	SQS	TD T0 – T1 [hh:mm]	TIU T2 – T3 [hh:mm]	Temp T2 – T3 [°C]	Diameter [mm]	Length [cm]
Median	3	3:01	1:10	0.5	1.5	17
Mean	3.1	3:29	1:50	1.5	2	20.7
Standard dev	1.2	1:45	1:49	2	1.4	11.8
Minimum	1	1:10	0:01	-1.7	0.7	8
Maximum	6	8:55	6:31	8	6	81

**SQS** Sample quality score*,* **T0** Euthanasia of the animal, **T1** Dissection of the nerve, **T2** Start of use in trials, **T3** End of use in trials*,* **TD** Time difference, **TIU** Time in use, **Temp** Temperature change

### Baseline assessment of the electrical properties

Amplitude, latency, threshold current (TC), and supramaximal voltage (SMV) were measured as basic signal parameters for all nerves with a positive signal response. Accordingly, a median amplitude of 5 mV (range: 0.2 mV – 25 mV), a median latency of 5.8 ms (range: 1.8 ms – 27 ms), a median TC of 0.2 mA (range: 0.1 mA – 1.2 mA), and a median SMV of 0.5 mA (range: 0.1 mA – 1.3 mA) were observed. Subsequently, the RLN was exposed to tensile forces with a median strength of 1.5 N (range: 0.5 N – 2 N) for a median time of sixty seconds (range: 30 s – 120 s). However, due to the high interindividual variability of the nerves' measured absolute signal values, all data were converted to percentages, with these baseline values serving as a 100% reference.

### Post-traumatic signal development and its influencing factors

**Fig. 2:**
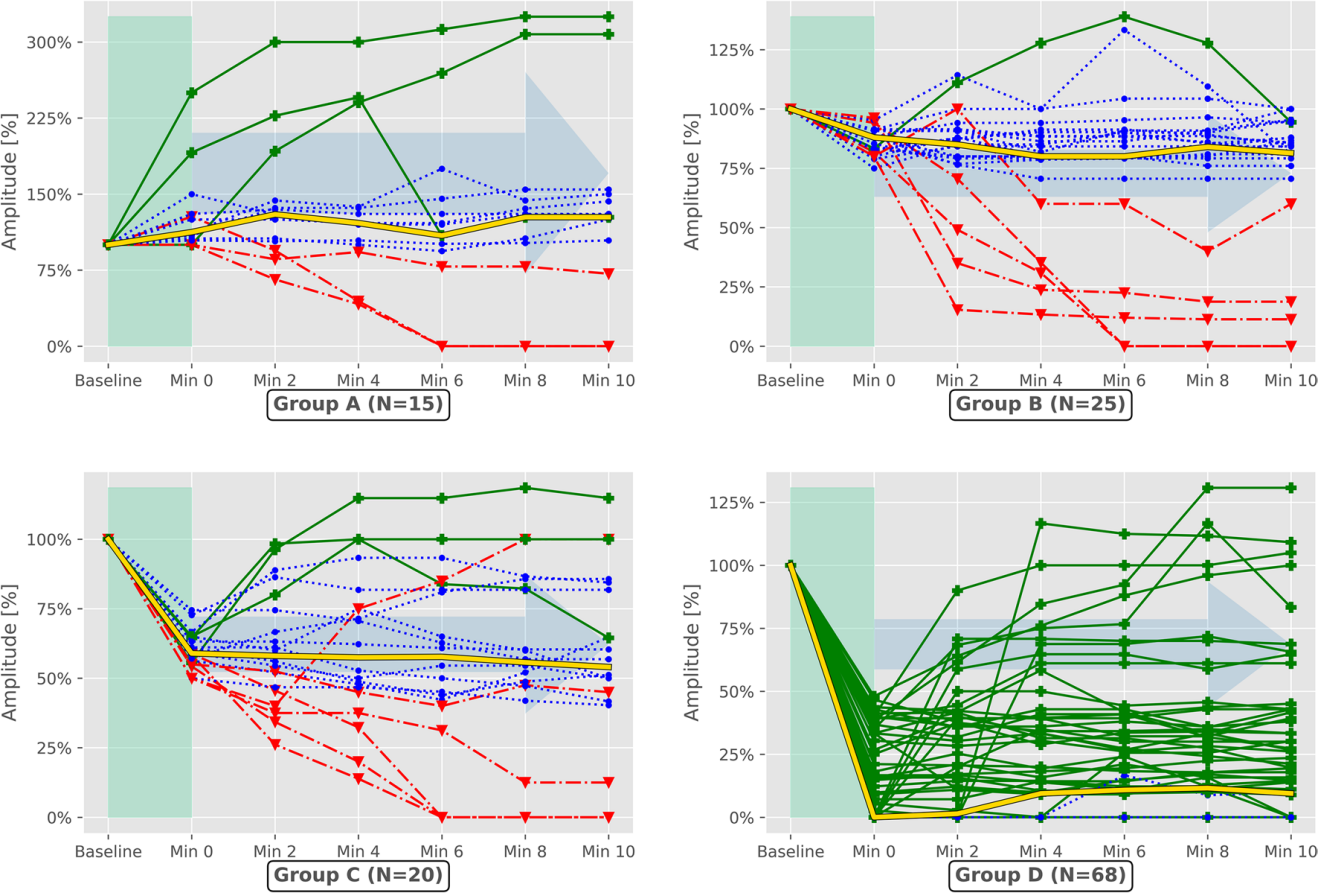
Illustration of the change in signal amplitude after stress exposure (Wilcoxon: p < 0.05) **Blue** Deviation of the median not exceeding 40%, **Red** Negative deviation exceeding 40% of the median, **Green** Positive deviation exceeding 40% of the median, **Yellow** Median of the respective subgroup **Group A** >  = 100% of baseline value, **Group B** < 100%, >  = 75% of baseline value, **Group C** > 75%, >  = 50% of baseline value, **Group D** < 50% of baseline value

Consistent with the absolute values before stress exposure, considerable variability was observed in the measured signal values following strain application. Although the amplitude of most nerves decreased, a subgroup of RLN exhibited a rise in amplitude (> 100%) compared to their baseline value (Fig. 2). We found a median post-stress amplitude of 42% (range: 0% – 250%), a median latency of 111% (range: 0% – 260%), as well as a median TC and SMV of 100% (range: 0% – 350%).

A highly significant difference in pre- to post-stress exposure amplitudes (p < 0.001) could be observed. Group A exhibited a median amplitude during all measurement time points (Min 0 – Min10) of 124% (range: 0% – 325%), Group B of 82% (range: 0% – 139%), Group C of 58% (range: 0% – 118%) and Group D of 9% (range: 0% – 216%).

A comparison of the signal development of nerves used just once, and those used repeatedly, showed no statistically significant difference in signal development. Furthermore, the amplitudes were separated according to their time difference (T0 – T1) and the time of use (T1 – T2) in two-hour intervals. Time in use did not influence signal strength, but a highly significant effect was observed with a time difference of more than six hours (p < 0.001). In addition, nerve length correlated with amplitude development (p < 0.05, r = 0.2) as did nerve diameter (p < 0.05, r = 0.5). A negative correlation between time difference and signal strength was observed after six hours (p < 0.05, r = -0.83).

Testing for confounders using a multivariate analysis confirmed a significant impact of nerve length (p < 0.05) on overall signal strength. In addition, Sample quality score, nerve length, and diameter were found to significantly affect groups B, C, and D (p < 0.05).

## Evaluation of the recovering RLN after LOS

Nerve exposure to noxious stimuli resulted in a complete LOS in 52 (40.6%) trials. Of those, 33 (63.5%) recovered from paresis with a positive amplitude. These nerves represented 12 cattle RLN (41.1% of all participating cattle nerves) and 21 (36.2%) pig nerves. Furthermore, 22 (66.7%) recovering RLN were derived from females and from 11 (33.3%) male animal nerves (Table 3).

**Table 3:** Overview of the physical characteristics of the recovering RLN

	SQS	TD T0 – T1 [hh:mm]	TIU T2 – T3 [hh:mm]	Temp T2 – T3 [°C]	Diameter [mm]	Length [cm]
Median	3	3:15	2:18	0.8	2	18
Mean	2.7	3:26	2:26	1.6	2.4	35.4
Standard dev	1.2	1:22	1:54	1.9	1.7	19.6
Minimum	1	1:31	0:20	-0.1	0.8	8
Maximum	5	8:30	6:07	8	6	81

**SQS** Sample quality score*,* **T0** Euthanasia of the animal, **T1** Dissection of the nerve, **T2** Start of use in trials, **T3** End of use in trials*,* **TD** Time difference, **TIU** Time in use, **Temp** Temperature change

**Fig. 3:**
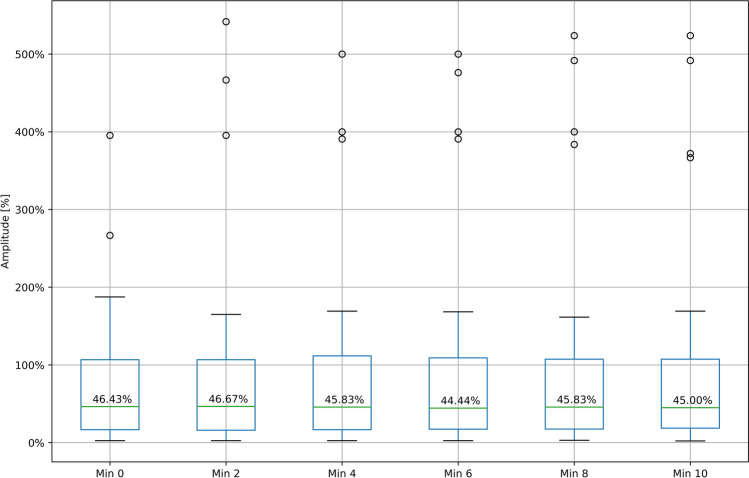
Amplitude development after signal recovery

Most of the LOS occurred immediately after nerve stress exposure (median: 0 min; range: 0 min – 80 min), whereas the time until signal recovery amounted to a median of 30 min (range: 2 min – 182 min). At recovery, a median amplitude of 46% (range: 2% – 395%), a median latency of 100% (range: 47% – 458%), a median TC of 100% (range: 17% – 450%), and a median SMV of 125% (range: 28% – 267%) were observed. However, no clear trend following signal recovery was evident, with some RLNs increasing up to 541% and others decreasing to 2% of their baseline value (Fig. 3). Since most nerves formed part of group D, this group also included the most recovering nerves (Table 4).

**Table 4:** Recovery characteristics of the respective groups, separated by PTA

Group	Median SOS [N]	LOS [N / %]	Recovery from LOS [N / %]	Median amplitude at recovery	Median time of recovery [min]
Group A [N = 15]	1	3 / 20%	1 / 33.3%	32%	61
Group B [N = 25]	1	4 / 16%	2 / 50%	67%	4
Group C [N = 20]	1.5	4 / 20%	4 / 100%	44%	14
Group D [N = 68]	2	42 / 61.8%	26 / 61.9%	46%	30.5

**SOS** Strength of strain, **LOS** Loss of signal, **PTA** Post-traumatic amplitude, **Group A** PTA >  = 100% of the baseline value, **Group B** PTA < 100%, >  = 74% of the baseline value, **Group C** PTA > 75%, >  = 50% of the baseline value, **Group D** PTA < 50% of the baseline value

## Assessment of the required tensile forces to induce LOS

**Fig. 4:**
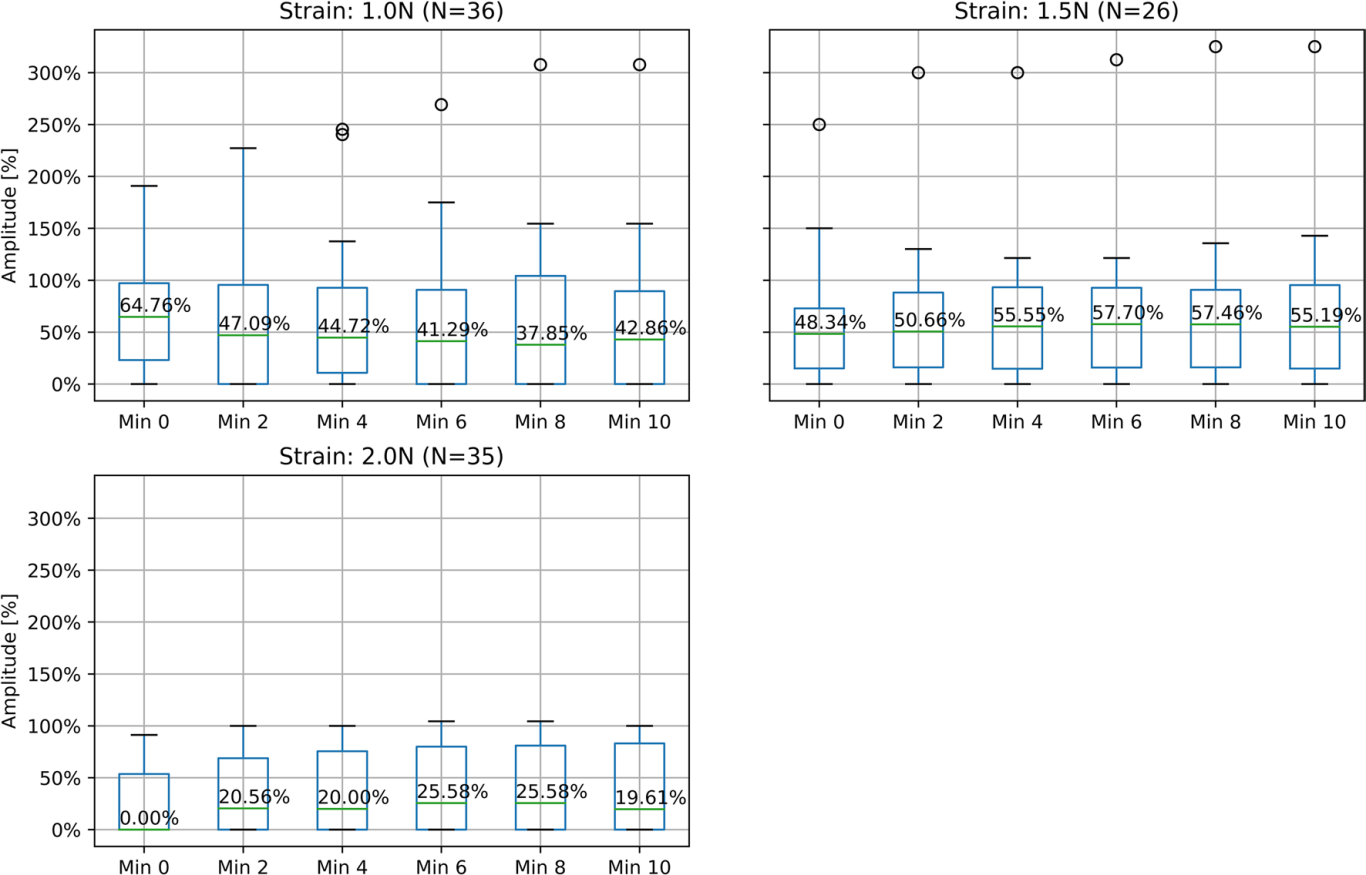
Progression of the nerve damage following 60 s stress exposure, separated by tensile force

To determine the required strength of strain (SOS) to provoke a LOS, 94 trials were performed. Nerves were divided into three new groups, based on the magnitude of strain applied. These included 1 N, 1.5 N, and 2 N for 60 s each (Table 5). The result was a significant (p < 0.05) decrease in amplitude (Baseline – Min 0) in all three subgroups. In addition, a significant difference (p < 0.05) between the amplitudes during the first four minutes (Min 0 – Min 4) of the 1 N and 2 N groups was found (Fig. 4).

**Table 5:** Injury characteristics for the different strain groups

SOS	Median post-strain amplitude	LOS [N]	Median time to LOS [min]
1 Newton [N = 34]	60% (Range: 0 – 130%)	13 / 38.2%	0 (Range: 0 – 6 min)
1.5 Newton [N = 25]	47% (Range: 0 – 150%)	6 / 24%	3 (Range: 0 – 10 min)
2 Newton [N = 35]	0% Range: (0 – 91%)	18 / 51.4%	0 (Range: 0 – 0 min)

## Discussion

Paralysis of the recurrent laryngeal nerve may result in adverse postoperative outcomes for patients, ranging from deglutition problems and dyspnea to dysphonia, including possible permanent loss of voice. However, the extent of manifestation depends on the severity of intraoperative injury [20]. Since the introduction of IONM for routine thyroid surgery, the identification rate of the RLN has steadily increased, allowing for a reduction of postoperative complications [10, 11]. Intraoperative nerve injuries are often caused by the many anatomic variations of the RLN, facilitating accidental injury during surgery, i.e., during thyroid retraction. Although recovery of RLN from paresis is possible, it can take up to a year to recover its function. Functional impairments after this time are considered permanent [10, 20].

This study aimed to demonstrate the feasibility of nerve conduction studies in our novel in-vitro neuromonitoring system. After RLN dissection, they were subjected to tensile strain with a median force of 1 N. Subsequently, the post-traumatic signal changes and their recovery were documented and analyzed.

Several in-vivo models analyzing RLN injuries and their regenerations can be found in the literature [10, 18, 21]. However, to the best of our knowledge, no in-vitro data using food industry cadaver nerves for nerve conduction analysis could be found. The present study thus proves the feasibility of the developed in-vitro system, offering several advantages. First, the associated cost is very low. Previous in-vivo systems used animals explicitly bred for clinical trials [10, 18], presumably representing a not inconsiderable cost factor. In contrast, the pharynges of cadavers from the food industry were available as abattoir waste at no additional cost. This allowed for a potentially more significant margin of error, as a larger sample size could compensate for the loss of individual RLNs. Second, the pharynges used were available almost every day, rather than on a fixed schedule – this thus reduced time pressure and allowed for easier planning and optimization of the model. Third, in-vitro experiments do not require approval by an ethics committee, thereby significantly increasing the system's adaptability. This allows for the rapid testing of new hypotheses without the need to apply for a new permit. In addition, existing proof of concept from in-vitro trials simplifies obtaining ethical approval for follow-up studies.

Due to the high anatomical comparability with humans, pigs were used in this study. In accordance with the German Animal Welfare Act [19], 297 V was applied to the animal's head and thoracic region during the sacrifice. In contrast, no electrical current was used during the cattle sacrificial process. As expected, this had a considerable impact on the percentage of RLN that later responded positively to in-vitro electrical stimulation. Indeed, 81% of the cattle nerves, as opposed to only 39% of the pigs' nerves, reacted positively to stimulation in the laboratory. This suggests the former to be more suitable for in-vitro experiments under these circumstances. The high electrical energy flowing through the pigs' bodies caused severe thermal and electrical tissue damage, as reflected in various stages of burn injury to the pharyngeal tissue. In addition, the removal of the pharynx from the animal carcass by non-surgically trained personnel at the abattoir caused further tissue damage of varying degrees.

Consistent with previous in-vivo studies, high inter- and intraindividual variability of the measured RLN amplitudes could be observed [18, 22]. Former studies have shown significant intrinsic variability in the RLN electrophysiological responsiveness even at low tensile stress levels, a phenomenon known as "weepy nerve" [18]. Using in-vitro nerves, we were able to observe the same pattern (Group A vs. Groups B, C, and D). Moreover, the study mentioned a higher variability of in-vivo RLN amplitudes compared with those of the vagus nerve. Possible explanations include variable moisture levels and nerve desiccation, besides electrode contact and position changes. Furthermore, anatomic interindividual differences in motor fiber arrangement within the nerve, i.e., concentric vs. eccentric, with respect to the stimulating electrode have been documented [22]. Thus, this study’s amplitude variability is presumably not exclusively attributable to ischemic, post-mortem neural changes. Instead, it may be influenced by the aforementioned environmental factors, confirming the possibility of studying nerve conduction disorders in an in-vitro environment.

Traction-induced neuronal damage has been reported to alter the electrophysiological properties of intra-axonal tetrodotoxin-sensitive sodium channels. Potentially, these contribute to a transient post-traumatic increase in transduction velocity. Moreover, a heterogeneous pattern of damage distribution within the injured axon has been observed [23]. Thus, possible explanations for the high variability in neuronal amplitudes in this study include 1) higher sensitivity and number of intra-axonal tetrodotoxin channels, 2) different intra-axonal connective tissue levels, resulting in varying degrees of protection from the noxious stimuli, and 3) the placement of needle electrodes within axons less affected by the tensile forces, due to the heterogeneous damage distribution pattern.

Previous studies have described an intraoperative decrease in RLN amplitude among the leading predictive factors for imminent vocal fold paralysis [10, 12, 15]. In addition, a significant decline in amplitude of more than 50%, or a 10% increase in latency, has been associated with potential neurological changes [24]. However, the period until nerve damage manifests is variable, and signal changes are often interpreted according to the visual perception of a positive or negative deviation. Consequently, early and subtle signal changes caused by acute nerve damage are often not immediately recognized intraoperatively, or detected too late [10]. This study revealed a further time-dependent loss of function during the ten-minute relaxation period, even after complete cessation of the noxious stimuli. For most of the nerves studied, the peak of their individual LOS was reached after six minutes. At this point, an approximate assessment of the nerve's health and a rough estimation of the signal progression was mostly possible. Thus, the time delay of the electrophysiological responsiveness has to be kept in mind when interpreting signals from continuous neuromonitoring.

To the best of our knowledge, the significance and effects of post-traumatically increased amplitudes have not yet been thoroughly investigated. Indeed, approximately 11.7% of all nerves examined in this study exhibited signs of post-traumatic hyperconductivity with transiently increased amplitudes. Surprisingly, complete LOS occurred in up to 20% of these nerves, despite the initially increased amplitude, a process we termed "weepy cry." Similar responses were observed in preliminary results of in-vivo trials (unpublished data). Nevertheless, further studies are needed to analyze the electrophysiological basis of this phenomenon, which is not yet entirely understood. These results underscore the importance of real-time feedback on nerve health via C-IONM. Care should be taken to monitor all amplitude variations since, even early, post-traumatically high amplitude values cannot definitively rule out further signal loss.

Nerves that show an intraoperative reduction in their signal amplitude may remain functional, a process known as nerve stunning [25]. Recovery may require several weeks to months [10]. The present study induced LOS in 40.6% of all investigated nerves. Besides 20% of Group A, these included 28% of nerves in Group B, 20% in Group C, and 61.8% of Group D. A tensile force of 2 N could be established as the threshold for paresis induction, following which up to 51.4% of all tested nerves exhibited LOS. In comparison, 38.2% of all nerves exposed to 1 N and 24% exposed to a force of 1.5 N showed signs of LOS. Therefore, in future workshops, surgeons could be trained to recognize a tensile strain threshold of around 1.5 N, to pause surgery to avoid potentially permanent nerve damage.

RLN recoveries after LOS have been described in the literature, given that neuronal continuity was maintained [10]. Cases of axonal transection are generally associated with a relatively poor surgical outcome [15]. Hence, the RLN in this study remained in their surrounding tissue complex. LOS was induced in 40.6% of all studied nerves. Up to 63.5% recovered with a positive amplitude after a median time of 30 min (range: 2 min – 182 min). The potential for signal recovery indicates that LOS was likely the result of an active, intrinsic traumatic tissue response instead of being caused exclusively by neuronal degradation. Future studies could investigate the mid to long-term effects of nerves recovering from acutely elicited, transient LOS, which might improve our understanding of this process's electrophysiological and anatomical background.

## Conclusion

The present study demonstrates that our newly developed in-vitro neuromonitoring system is a viable alternative to in-vivo animal studies. It provides a rapid and cost-effective analysis of subtle, post-traumatic neural signal changes, as the mechanisms leading to signal loss are not yet fully understood. Future studies could use this model to analyze potential nerve-protective measures.

After exposing the RLN to tensile forces of a median of 1 N, a time-dependent progressive loss of function was observed during the ten minutes following cessation of strain. Signal recovery after a further resting period indicates, that these changes presumably are attributable to intrinsic neural processes and not exclusively post-mortem neural cell death. High measured amplitude values are considered to indicate intact nerve conduction. Immediately following cessation of strain, a hyperconductivity with increased signal amplitudes respective to their baseline values was found in a subgroup of the examined nerves. Surprisingly, complete paralysis subsequently occurred in up to 20% of this subgroup, a phenomenon we termed the "weepy cry." Nevertheless, its pathophysiological basis remains unclear. Preliminary data from in-vivo studies have confirmed these observations (unpublished results). Thus, these findings require further electrophysiological investigations and highlight the importance of real-time intraoperative neuromonitoring to avoid inadvertent nerve injury.

## Data Availability

The data supporting the findings of this study are available upon reasonable request. Requests for access to the data can be made to the corresponding author, and will be considered in accordance with applicable data protection and privacy regulations.
